# Measuring achievement emotions questionnaire for physical education (AEQ-PE): a confirmatory study in Malay language

**DOI:** 10.1186/s12889-021-11869-4

**Published:** 2021-10-07

**Authors:** Mohamad Fadil Ibrahim, Garry Kuan, Hairul Anuar Hashim, Nurul Azuar Hamzah, Yee Cheng Kueh

**Affiliations:** 1 Exercise and Sports Science Programme, School of Health Sciences, Universiti Sains Malaysia, 16150 Kubang Kerian, Kelantan Malaysia; 2 Institit Pendidikan Guru Malaysia, Kampus Sultan Mizan, Besut, Terengganu Malaysia; 3 Department of Life Sciences, Brunel University London, Uxbridge, UB8 3PH UK; 4 Biostatistics and Research Methodology Unit, School of Medical Sciences, Universiti Sains Malaysia, 16150 Kubang Kerian, Malaysia

**Keywords:** Achievement emotion, Physical education, Enjoyment, Pride, Anger, Anxiety, Hopelessness, And boredom

## Abstract

**Background:**

This study aimed to verify a translated Malay version of the Achievement Emotions Questionnaire for Physical Education (AEQ-PE) by assessing the level of achievement emotions in six constructs among the Malaysian primary school pupils using the Confirmatory Factor Analysis (CFA).

**Methods:**

A total of 607 Malay pupils, comprising 240 (39.5%) boys and 367 (60.5%) girls aged between 10 and 11, were recruited from 10 schools to answer the questionnaire, which measured their views on 24 items through a five-point Likert scale. The AEQ-PE was translated into Malay language (AEQ-PE-M) using forwarding to backward translation techniques. Certain phrases were adopted in accordance with the local culture and vocabulary appropriate for primary school pupils. CFA was performed using the Mplus 8.0 software, and the final model demonstrated high reliability in terms of the composite reliability and Cronbach’s alpha.

**Results:**

Analysis of the CFA showed an acceptable fit indices in CFI (0.936), TLI (0.926), RMSEA = 0.039 (90% CI, 0.034, 0.045) and SRMR (0.049) of the AEQ-PE measurement model. All of the items in the original AEQ-PE version were retained and deemed suitable for Malay primary school pupils.

**Conclusion:**

The AEQ-PE-M with 24 items was a suitable tool for measuring the level of school children’s involvement in determining achievement emotions and their motivation towards physical education.

## Introduction

Emotions are an important factor in influencing student motivation and achievement, especially in terms of learning, performance improvement, identity development, and health [1–4]. Theoretically, instruments to “measure” or gauge emotions are important in assessing individual feelings because different situations experienced by each person would influence the individual’s response towards a certain task like learning [5]. A key requirement in assessing emotional tendencies is questionnaires that contain the right items to facilitate the respondents’ answers about their feelings [6]. The selected items in this study’s questionnaire aim to assess the extent of students’ emotional inclination towards their academic environment [7–9]. Studies have also assessed the quality of instruments related to psychometry and the role of emotional predisposition in the learning process [10, 11]. Therefore, theoretically, researchers need specific questionnaires to analyse the function of evaluating emotions in educational practice [12]. This study involves a preliminary investigation into the emotions related to achievements in Physical Education, which are relevant in testing all measurement scales that have been used in the Achievement Emotions Questionnaire in Physical Education (AEQ-PE) assessment.

The AEQ-PE is an integrative questionnaire model for assessing emotionally related behavioural changes that are developed based on various psychological aspects [10]. It has been widely used to measure emotions that influence behaviour [10]. Emotional achievement occurs in different academic environments. The fun of learning may be different for each of the students in enjoying the learning challenges. Some students may be enthusiastic before the start of class, while some do not like to attend class and some others have feelings of frustration after class. Therefore, the measure of emotional inclination varies among students because the emotions experienced by individuals are not the same even in various similar situations. The questionnaire is divided into three parts to assess the situation before, during, and after attending a class, using six constructs that encompass the students’ feelings, namely enjoyment, pride, anxiety, anger, hopelessness, and boredom. The validity process of the AEQ-PE through a psychological approach will increase student motivation, as well as influence their performance towards better academic and emotional achievements [13]. The validity of instruments related to the theory of emotional predisposition is used [14, 15] by providing an integrative approach to analyse the various emotional states experienced in the context of other academic and social achievements, which are more dominant towards students [13].

Emotional achievement occurs in different academic environments [16–18]. The construction of the AEQ-PE through the achievement assessment model is meant to test the respondents’ behavioural and psychological aspects. The first stage of this study is to look at the validity and reliability of the AEQ-PE that has been translated into Malay language and tested on Malay primary school pupils. Next, the researchers conduct an empirical analysis to explain the statistics for each item and scale related to the reliability and validity (internal and external) of the instrument. Therefore, theoretical tools are needed to analyse the function to see the differences, especially those related to local culture in assessing the emotions that can be practiced in education, especially in the framework of primary school pupils in Malaysia [19].

The original AEQ assesses nine aspects (enjoyment, hope, pride, relief, anger, anxiety, hopelessness, shame, and boredom) and is used comprehensively to assess emotional achievement in general. The use of the AEQ-PE in this study has been modified to include physical activity constructs, such as hope and relief [8, 20]. Learning fun may be different for each student as they face and overcome challenges in the process [13, 21]. Some students may be enthusiastic to attend class, while others are already disinterested before the lesson can even begin. And after class, if expectations are not fulfilled, some may end up feeling frustrated [18, 22]. Therefore, there is a need to measure emotional achievement based on different tendencies among students because the emotions experienced by every individual are not the same, even when going through very similar situations [23]. Emotional measures for PE do not always measure in relation to the learning process or examination situation, but more specifically are to measure student achievement in relation to the class [14]. This study was to distinguish emotional states with variables that influence feelings and attitudes in PE consistently when associated with physical activity involvement in school [24, 25]. Therefore, this study aimed to confirm the six-factor structure of the Malay version of the AEQ-PE and provide the construct validity and reliability of the scale.

## Methods

### Study design

This quantitative study was conducted at the primary schools in Jerteh Terengganu, Malaysia. For the AEQ-PE-M, a cross-sectional study design was used. Data collection took place between June 2019 to Jan 2020, at ten primary schools in the district of Besut, Terengganu, Malaysia. This study determines the fit of the hypothesized measurement model to the data obtained and the reliability and validity of the model construction using confirmatory factor analysis (CFA). Those who volunteered for this study completed the questionnaire via Google Forms in the school computer lab with the assistance of Information and Communication Technology teachers and then was send directly to the researchers. All participants voluntarily completed the AEQ-PE-M and submitted their parental consent and their informed consent form (assent form). Participants who had not acquired parental consent were excluded.

### Participants

A total of 619 Year 4 and 5 pupils aged between 10 and 11 were randomly chosen from 10 primary schools in Besut district, Terengganu, Malaysia, using a computer-generated random number. The inclusion criteria included those who participated in physical activities. All participants were given the Malay version known as the AEQ-PE-M, adapted from the AEQ-PE (i.e., Fierro-Suero et al. [8]). The response rate was 98.5%. There were 12 incomplete questionnaires, which were excluded. When conducting the confirmatory factor analysis (CFA), a large sample size would generally produce more robust solutions and likely to be replicable [26]. Based on Hair et al. [27] the sample size required for CFA would be about 500 subjects for large numbers of constructs. Therefore, the sample size of 607 in the present study was deemed sufficiently large for the confirmatory study.

### Measures

#### Achievement emotions questionnaire (AEQ-PE)

The AEQ-PE was developed by Fierro-Suero et al. [8]. This questionnaire was designed to measure achievement emotions experienced by pupils. The AEQ-PE aspects included 24 items that required pupils to explain how they felt when undertaking a subject. Each item contained measurements related to affective, cognitive, motivational, physiological, and emotional components. The questionnaire comprised six constructs (enjoyment, pride, anger, anxiety, hopelessness, and boredom), each construct consisting of four items. A five-point Likert scale (1-strongly disagree to 5- strongly agree) was used to record the pupils’ responses to the items. The internal consistency measured by Cronbach’s alpha for all six constructs were acceptable, ranging from .72 to .94 [8].

#### Questionnaire translation process

The original AEQ-PE questionnaire was translated into Malay by using standard procedure of forward and backward translation recommended by Brislin and based on previous studies involved questionnaire translation from English to the Malay language [28–30]. The reversal procedure is used to ensure that it is related to the content of the item equality. First, two content experts reviewed the translated English version into Malay, ensuring that the translation made by the researchers retained the meanings and considered the level of children’s vocabulary and understanding. Second, two sports science lecturer experts determine the appropriate level of the Malay language is adjusted according to the level of cognitive that is appropriate to the child and then subsequently translated back into English.

Next, two bilingual Malay language lecturers back-translated the Malay version into English, and both versions were compared for any difference in meaning. Thirdly, three lecturers and three teachers with backgrounds in sports psychology, health psychology, physical activity, and sports science with a focus on physical education reviewed the English translation from Malay and the Malay translation from English and compared each item to the original English version. All the six members in the panel had over 10 years of experience in their respective fields and were bilingual and fluent in Malay and English language. Additionally, they also evaluated whether the content was consistent with Malaysian culture and ensure that it does not deviate from the original meaning and purpose of the survey. Finally, a pre-testing of the final version AEQ-PE-M was conducted with 30 students at Sekolah Kebangsaan Bukit Kenak in Jerteh, Terengganu for its suitability for the study population.

#### Procedures

The research was a performance in compliance with the Declaration of Helsinki and was approved by the Universiti Sains Malaysia Human Research Ethics Committee (USM/JEPeM/19090518). Study approval was obtained from the Malaysian Ministry of Education, the Terengganu State Education Department, and headmasters of the respective schools. A research information sheet on this study was distributed to the pupils’ parents or guardians, and written informed consent was obtained both from the parents and the pupils before beginning the study. The pupils were told that their participation was voluntary and that they could withdraw from the study at any time. Questions relating to personal identifiers were not included in the questionnaire. The questionnaire was administered to the pupils online during school hours via Google Forms in the schools’ computer lab with the help of teachers, who facilitated the sessions and ensured that pupils submitted their answers. The students took approximately 20 min to complete the questionnaire. Once they finished, they were given a certificate as a token of appreciation.

#### Data analyses

Descriptive statistics and internal consistency based on the Cronbach’s alpha value were conducted using SPSS 27.0 (IBM Corp, Armonk, NY, USA). For reliability based on Cronbach’s alpha, the acceptable value was 0.70 and above [27].

The measurement CFA model of the AEQ-PE-M was tested using Mplus 8.0 (Muthen & Muthen, Los Angeles, CA, USA). The assumption of multivariate normality was checked using Mplus and we found it was not met (Mardia multivariate skewness and kurtosis test with *p*-values less than .05). Therefore, the MLR estimator was then used in the subsequent CFA analyses. The initial hypothesized measurement model consists of 6 latent variables (subscales of the AEQ-PE-M) and 24 observed variables (items in the AEQ-PE-M). Factor loadings of .40 and above, with significant *p*-value were used as a guide to retain or remove the items from the measurement model [31, 32]. Modification index (MI) was referred for model re-specification including adding items’ residual correlation in the model. Model re-specification was done after theoretical support was carried out by the researchers. Evaluation of fitness has been carried out each time the model has been re-specified, or a problematic item has been removed. Multiple fit indices can be used to examine the model fitness [27, 33]. The fit indices used in this study and their recommended cut off point were: comparative fit index (CFI) and Tucker-Lewis Coefficient (TLI) indices with the desired value greater than 0.92; and root mean square error of approximation (RMSEA) and standardised root mean square residual (SRMR) with values lesser than 0.08.

After the best-fit measurement model was established, the six factors were assessed for composite reliability (CR) and discriminant validity, CR was calculated based on Raykov’s method to measure the reliability of the CFA measurement scale [34]. The minimum acceptable value of CR is .60 and above [27]. Discriminant validity is used to analyse the degree to which one factor is distinct from the other factors [33]. If the correlation coefficient between factors is not too high ≤.85, then the validity of discrimination can be identified [20].

## Results

Table 1 shows demographic information and the frequency of students. The total number of respondents was 607 participants: 240 (39.5%) were male, and 367 (60.5%) were female. When considering respondents by age categories, a total of 186 (30.6%) respondents were aged 10 years and 421 (69.4%) respondents were aged 11 years.

**Table 1:** Demographics and stages of change of school children (*n* = 607)

Demographic	Frequency (%)
Gender	
Male	240 (39.5%)
Female	367 (50.5%)
Age: 10 Years	186 (30.6%)
11 Years	421 (69.4%)
Ethnics: Malay	607 (100%)

### Construct validity and reliability

**Fig. 1:**
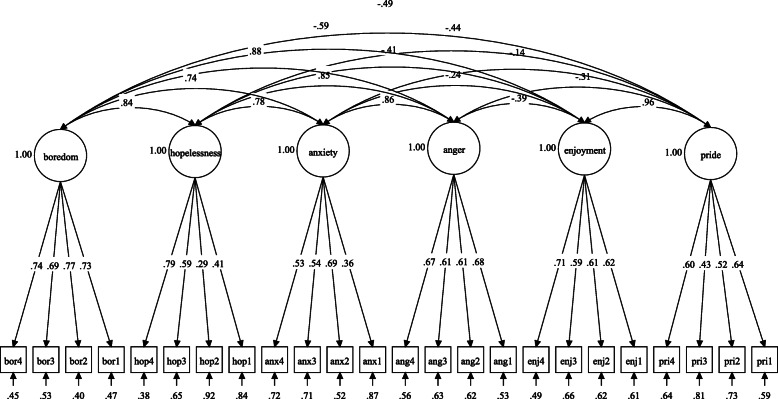
Illustrates the CFA diagram of the AEQ-PE-M

The results of the analysis showed a good fit to the six-factor model based on the fit indices (CFI = 0.936, TLI = 0.926, RMSEA = 0.039 (90%CI, 0.034, 0.045), SRMR = 0.049). The standardized factor loadings were statistically significant (*p* < 0.001) and ranged from 0.29 to 0.79 (see Table 2). Figure 1 illustrates the CFA diagram of the AEQ-PE-M. There were two items with factor loading less than the recommended value (< 0.40). The items were kept in the model because we aimed to maintain the original content of the questionnaire, also considering a small number of items per factor (4 items per factor) and besides, the fitness of the model was acceptable without removing the low loading items.

**Table 2:** Standardized factor loading of the AEQ-PE-M based on CFA

Factors/items	Factor loading	Cronbach alpha	Composite reliability
**Factor 1: Pride**			
I am proud to be able to keep up with the physical education class.	0.637	0.64	0.63
I am proud of my participation in a physical education class.	0.517		
I think that I can be proud of what I know about physical education.	0.435		
Because I take pride in my accomplishments in physical education, I am motivated to continue	0.597		
**Factor 2: Enjoyment**			
I am motivated to go to the physical education class because it is exciting.	0.623	0.73	0.73
I enjoy being in the physical education class.	0.615		
I feel excited about being in a physical education class, practicing what the teacher suggests.	0.586		
I am glad going to the physical education class paid off.	0.715		
**Factor 3: Anger**			
I feel anger welling up in me during the physical education class.	0.683	0.74	0.74
Because I am angry, I get restless in the physical education class.	0.615		
Thinking about all the useless things I have to learn in physical education, annoys me.	0.612		
After the physical education class, I am angry.	0.665		
**Factor 4: Anxiety**			
I worry that the things I have to do in physical education classes might be too difficult.	0.357	0.63	0.61
I feel nervous in the physical education class.	0.692		
I get scared that I might say/do something wrong in the physical education class and would rather not say/do anything.	0.541		
When I do not understand something in the physical education class, my heart races.	0.528		
**Factor 5: Hopelessness**			
It is pointless to prepare for the physical education class because I am bad at it anyway.	0.405	0.61	0.61
Even before entering the physical education class, I know I will not get it right.	0.287		
I would rather not go to the physical education class because it is impossible to perform the exercises correctly.	0.593		
I have lost all hope of doing physical education activities effectively	0.785		
**Factor 6: Boredom**			
I feel like leaving during the physical education class because it is so boring.	0.731	0.82	0.82
I get bored during the physical education class.	0.772		
The physical education class bores me.	0.689		
I find the physical education class fairly dull.	0.742

The reliability of the questionnaire was computed based on two methods: internal consistency based on Cronbach’s alpha and composite reliability based on the CFA model. The reliability value for each factor was reported in Table 2. The Cronbach’s alpha for the six factors was ranged from 0.61 to 0.82 and the composite reliability were ranged from 0.61 to 0.82. The reliability of the six factors model and 24 items were acceptable.

Figure 1 illustrates the final CFA model in a diagram. Several factors had correlation more than the recommended value of 0.85 for discriminant validity. The factors were enjoyment and pride, hopelessness and anger, boredom and anger, anxiety, and anger. These highly correlated factors were not combined as single factor because researchers decided to maintain the theoretical framework of the original AEQ-PE.

## Discussion

The AEQ-PE has been extensively translated and used to assess emotional achievement in six main areas (i.e., enjoyment, pride, anxiety, anger, hopelessness, and boredom) in physical education among different populations [8, 19, 21]. Researchers have used the questionnaire as a tool to test the students’ emotional achievement before, during, and after class. The AEQ-PE-M, which was the AEQ-PE translated from English to the Malay language, could fill the gap and serve as a tool to identify emotional achievement of rural Malay primary school pupils that used the latter language as the lingua franca, though English was commonly used in Southeast Asian countries [20]. The validation study aimed to confirm whether the measurement model of AEQ-PE-M with six-factors could be well adapted to pupils of the same age in Malaysia. The results of the CFA analysis clearly showed that the validity and reliability of the AEQ-PE-M could be considered suitable for use in primary school students aged between 10 and 11.

Based on this empirical study, the emotional achievement was assessed in the physical education of pupils through motives of involvement, especially relating to the pleasure of coming to school. The AEQ-PE measured a general correlation related to emotional achievement involving three different classroom situations, in addition to being associated with physical activity and a tendency to attend school [11, 14]. Therefore, many researchers had attempted to translate the AEQ-PE into their respective local languages to gauge the achievement motivation and emotion of pupils in line with the local culture and vocabulary to get a clearer understanding and accurate response.

In this study, the validation of the original English version of the AEQ-PE factor had been proven to be reliable, valid, and stable over a certain period based on previous studies [14, 19]. The studies on validity and reliability models were necessary to produce consistent questionnaires, which required greater modelling by discarding items with a low factor loading if any [35]. In this context, the AEQ-PE-M had been assessed and was observed to demonstrate greater model and validity values after the exclusion of items showing a low factor loading. Many studies using the AEQ-PE had shown good performance among students in secondary schools, but such results had yet to be observed in primary school pupils. Therefore, in this study, the items in the AEQ-PE-M were tailored to the culture and understanding of Malay primary school pupils.

Previously, we found no specific instruments to measure the level of enjoyment and satisfaction experienced by Malay students in learning physical education. This instrument could be considered valid because the questions were suitable to determine the extent to which pupils experienced enjoyment, especially in physical education. Researchers, sport psychologists, and teachers could consider this questionnaire a useful and moderate tool to be applied routinely in measuring and monitoring the psychological effects of achieving emotional satisfaction among primary school pupils in Malaysia [36–38]. The capability of using the AEQ-PE-M to study the role played by emotions in academic performance, pupil engagement, as well as motivation for the physical activity made the questionnaire all the more interesting [8]. Therefore, improvements to lessons could be made through strategies that increased pleasure and satisfaction in learning, which in turn, would increase pupils’ participation and enthusiasm in physical activities. If well-integrated, this could become a useful value-added tool in daily training to achieve emotional awareness of this vast aspect of life.

There were some limitations in this study. The study participants were limited to one district in Malaysia which could not be representative to all students in Malaysia. Therefore, future studies should include larger samples of study population in Malaysia. This could be done by involving more schools in different districts and states in Malaysia. Multistages cluster sampling method could be applied to select numbers of schools from all the schools listed in Malaysia. By doing this, the study samples could be generalised and represent the whole Malaysia’s primary school pupils. In addition, primary school pupils from different ethnic’s background such as Chinese and Indian should be included in the study samples. The participants may also answer the question in a socially acceptable manner. To overcome this limitation, researchers had put in efforts to explain to students to give their honest answers during the study briefing session. Future study should also include additional validation test such as criterion validity by comparing the AEQ-PE-M with other established questionnaire measuring the achievement emotions experienced by schools’ students. Besides, measurement invariance test should be conducted in future to investigate whether the AEQ-PE-M is equivalent among gender and different ethnic groups in Malaysia. This will provide further evidence on validity of the scale. Although the Cronbach’s alpha for AEQ-PE-M were lower than the previous study done by Fierro-Suero et al. [8], there were still within the acceptable range (> 0.60) as suggested by some literature [39, 40]. However, we would suggest future research should consider improving the Cronbach’s alpha by adding new items or revised some items with low factor loading.

## Conclusion

Positive emotions experienced by students can increase the desire to learn as well as encourage students’ interest and involvement in PE. However, the implications of negative emotions on students on PE still need to be clarified and detailed to provide a clearer picture related to the class [41–43]. In conclusion, this study showed that items in the English version of the original AEQ-PE could support the validity and reliability of the AEQ-PE-M among primary school pupils in Malaysia. Therefore, with the AEQ-PE-M, education policymakers, researchers, sports psychologists, and teachers would have an instrument with accurate translation and reliability to do so. Moreover, the AEQ-PE-M might even obtain unique emotional profiles experienced by pupils in more practical situations. Positive emotions in PE are emotional states that get a lot of attention because they promote student interest and engagement. However, the implications of negative emotions on students towards PE are still poorly explained in relation to class.

## Data Availability

The dataset used and analysed during the current study is available from the corresponding author on reasonable request.
